# Furthering the Continental Drift Speciation Hypothesis in the Pathogenic *Cryptococcus* Species Complexes

**DOI:** 10.1128/mSphere.00241-17

**Published:** 2017-06-14

**Authors:** David M. Engelthaler, Wieland Meyer

**Affiliations:** a Translational Genomics Research Institute, Flagstaff, Arizona, USA; b Molecular Mycology Research Laboratory, Center for Infectious Diseases and Microbiology, Marie Bashir Institute for Emerging Infectious Diseases and Biosecurity, Westmead Clinical School, Sydney Medical School, Westmead Hospital, The University of Sydney, Westmead Institute for Medical Research, Sydney, Australia; Duke University Medical Center

**Keywords:** *Cryptococcus*, speciation, *C. gattii*, Pangea

## LETTER

We read with interest the continental drift hypothesis by Casadevall et al. (1) as a possible speciation driver within the *Cryptococcus* species complexes, adding greatly to the ongoing discussion of speciation between these complexes (2, 3). We further propose that this mechanism may also have had speciation effects within these complexes, most notably within *Cryptococcus gattii*, where at least four major molecular types/species are recognized (VGI, VGII, VGIII, and VGIV). It is likely that with hundreds of thousands of single nucleotide polymorphism (SNP) mutations separating the major *C. gattii* molecular types (4), their temporal separation is in the tens of millions of years (5, 6). *C. gattii* was originally considered a tropical pathogen, being endemic to Australia, Asia, Africa, and South America, with random cases appearing in North America and Europe (7, 8); it is either nonendemic or newly endemic to these latter continents due to global movement of *C. gattii* microhabitats, such as eucalypts (e.g., *Eucalyptus camaldulensis*) and Douglas fir (*Pseudotsuga menziesii*) (9, 10).

**FIG 1:**
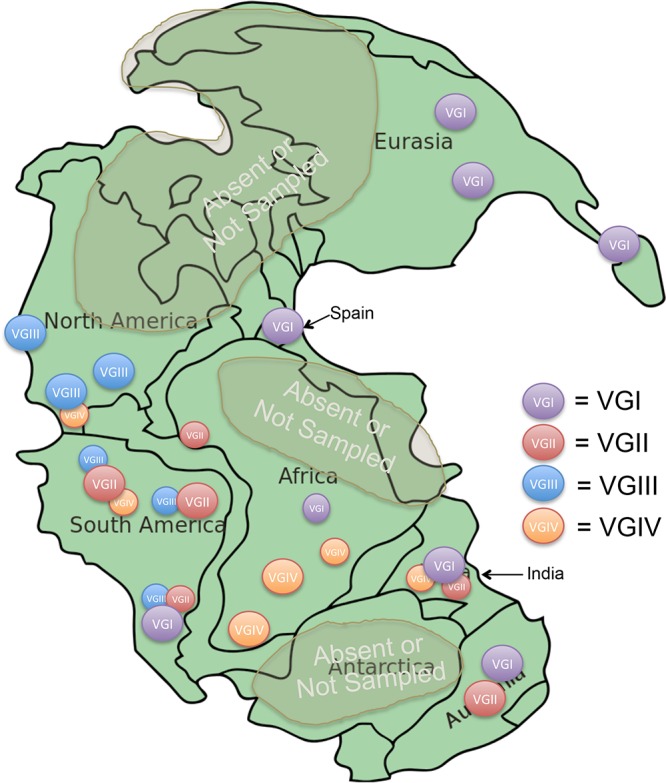
A Pangea representation of present day geographically dominant *C. gattii* populations. Note that nonendemic isolations and more recent emerged populations are not displayed. (Image adapted from https://commons.wikimedia.org/wiki/File:Pangaea_continents.png.)

Based on global genotype data, we noted that the molecular types appear to have hemispheric and continental associations (Fig. 1). Asian *C. gattii* isolates are predominantly VGI, with a low-level VGII presence in most Asian countries (11). VGIV has only been reported in India (12), and VGIII appears to be absent in Asia, except in Thailand (13). Conversely, VGIV is the dominant *C. gattii* molecular type in Africa (11). Only VGI and VGII have been identified in Australia, one of the first places where *C. gattii* was found in the environment and therefore previously considered to be a possible birthplace of *C. gattii* (14, 15). There are limited to no reported findings of VGIII in Australia or Africa. In Europe, except for Spain, where a significant number of VGI strains have been identified, isolates are largely clinical or associated with nonnative trees (9, 11). Most United States cases are traveler associated or are due to recent translocations of the fungus from regions of endemicity. Beyond the emergence of VGII out of Brazil into the Pacific Northwest (4, 16, 17), VGIII emergent events have occurred in California (18) and the southeastern United States (19), and VGIII also dominates among isolates from Mexico (20, 21). South America has a high degree of disease, primarily in three countries: Brazil and Columbia have predominantly VGII (22), with lower levels of VGI and VGIII (17) and, rarely, VGIV, whereas isolates in Argentina are primarily VGI (11). Numerous isolations from native Amazonian rain forest trees far removed from human interactions suggest an ancestral location for *C. gattii* (23–25).

Of interest are possible remnant populations from prior contiguous Pangea regions. For example, India was a contiguous landmass with southern Africa (R. W. Schlische; http://www.rci.rutgers.edu/~schlisch/103web/Pangeabreakup/breakupframe.html). It is possible that a common ancestor to VGIV was endemic to such a region prior to the break off of the Indian Subcontinent. Other interesting phylogeographic features include African VGII being found only in Senegal, a previous land partner with Brazil (17), and the presence of apparently endemic European *C. gattii* primarily only on the Iberian Peninsula, Europe’s Pangea connection to Africa (R. A. Krulwich; http://www.npr.org/blogs/krulwich/2013/09/12/221874851/a-most-delightful-map.

This is not to suggest that the above endemic foci are all due to separation of contiguous endemic populations during the breakup of Pangea, nor does this represent an exhaustive listing of all isolations of *C. gattii* around the world. It is however an additional viewpoint in favor of the continental drift dispersal hypothesis.
